# Miscellaneous Items

**Published:** 1855-05

**Authors:** 


### MISCELLANEOUS ITEMS.

Cockroach Riddance.—The Scientific American of New York, which fails not in its weekly appearance to give some little family recipe, *which may be relied on*, of more practical utility than the two dollars which would pay for its yearly subscription, vouches for the fact that

"*Common red wafers, scattered about the haunts of cockroaches, will often drive away, if not destroy them.*"

These wafers, like candies, are colored red by oxyde of lead; a most deadly poison, and so is the acetate of lead, or sugar of lead, as it is sometimes called, on visiting cards, which being a little sweetish, has been known to destroy young children to whom they were handed, to be amused with. Fashion for once acts sensibly in discarding glazed cards, using instead, *Bristol board*, more pliant, less cumbersome, and really more delicate. And while we are speaking of one of the pests of housekeepers, it may be well to know,

How to get rid of Rats, old, young, and middle aged, with the shortest possible suffering to them, and with small probability of their dying in their holes, or other uncomeatable places.

Spread a level teaspoon of flour or corn-meal on a chip or small piece of dirty board, sprinkle over this half a grain of strychnine; it kills the rat before he can get to his nest.

It would be wrong to let this statement pass, in a journal like this, without cautioning the reader that strychnine is a fine white powder, much like flour, made from the seeds of a fruit which looks like an orange, growing on a moderate sized tree in the East Indies, in the Island of Ceylon and neighboring islands. A sixth of a grain of pure strychnine will kill a dog in half a minute. One grain, which would easily lie on a three cent piece, or even less, may prove fatal to a man. Hence the reason for not mixing more than half a grain at a time, and by putting it on a chip, or dirty board, it would not be likely that children would taste it, although the mixture with flour, looks very much like white pulverized loaf sugar. As it is such a deadly and instantaneous poison, no more than half a grain should be purchased at a time; it should not be allowed to pass out of the hands of the head of the family for a single moment. The mixture should be placed in a room the last thing at night, the door locked, the key put in the pocket, and removed the first thing in the morning, by throwing chips and all into the fire, washing the hands well after doing so, as also after first mixing it, for a great deal less than a grain would kill a man, if it happened to fall on a sore or cut finger.

---

A New Wrinkle.—It is said to have been satisfactorily demonstrated that every time a wife scolds her husband she adds a new wrinkle to her face! It is thought that the announcement of this fact will have a most salutary effect, especially as it is understood that every time a wife smiles on her husband it will remove one of the old wrinkles! Mr. Caudle is delighted with the discovery, and anticipates sunshine the year round, as Mrs. Caudle has an unquenchable desire to appear young and handsome, and mourns deeply over the rapid departure of her youthful charms. Poor, curtain-lectured husbands are looking up.

---

Punishment of Babbling Women in "Old Virginny."—As late as 1792, the following act is said to have been passed by the Legislature of Virginia: "An Act for the punishment of scandalous persons.—Whereas, many babbling women slander and scandalize their neighbors, for which their poor husbands are often involved in chargeable and vexatious suits and costs in great damages.—Be it therefore enacted by the authority aforesaid, that in actions of slander, occasioned by the wife, after judgment passed for damages, the woman shall be punished by ducking; and if the slander should be so enormous as to be adjudged at greater damages than five hundred pounds of tobacco, then the woman to suffer a ducking for each five hundred pounds of tobacco adjudged against the husband, if he refuses to pay the tobacco."

---

The Proportion of Deaf and Dumb in Europe. — It appears that the proportion of deaf and dumb in Ireland is one in every 1,593 inhabitants; in the Duchies of Luxembourg and Wurtemburg, and the kingdoms of Tuscany, Bavaria, Belgium, and Holland, one in every 2,209; in Sardinia, Norway and parts of Switzerland, one in every 642. In some of the Swiss cantons the average is as high as one in every 506—more than seven times as great as in Ireland.

---

Square Feet in an Acre.—An acre contains 43,560 square feet.

A plot of ground $208\frac{3}{4}$ feet square is very near an acre, being just 1-16 of a rod over. A nearer approximation is 208 feet and $9\frac{1}{2}$ inches. The square of this number differs less than a foot from an acre, being 43,559 1-6 feet.

A plot of ground 12 rods 10 feet and $8\frac{1}{2}$ inches square, is an acre. For ordinary purposes it will answer to take a plot $12\frac{2}{3}$ rods square, which will give 160 2-5 rods, 160 being an acre.

An acre is contained in a plot 3 by $53\frac{1}{3}$ rods; or 4 by 40; or 5 by 32; or 6 by $26\frac{2}{3}$; or 7 by 22 6-7; or 8 by 20; or 9 by 17 7-9; or 10 by 16; or 11 by 14 6-11; or 12 by $13\frac{1}{3}$.

---

Causes of Insanity.—In the report made by Dr. Blanchard, of the Kings County Lunatic Asylum, some singular features are developed as to the causes operating to produce insanity. The relative liabilities of the two sexes to be affected by causes operating upon the affections are also exhibited. The loss of a daughter caused the insanity of one woman, and the loss of a wife produced the same result in one man. One man and two women were crazed by disappointed love, one man and three women by jealousy, one woman by the desertion of her husband, and one man by domestic difficulty; three men and three women became insane by uncontrollable temper, one woman by hatred of her step-mother, three men and three women by religious enthusiasm, twelve men and eleven women by dissipation.

---

Boiled Plum Pudding.—Take one pound of good suet, cut it into small pieces, and add one pound of currants and one of stoned raisins, eight eggs, one nutmeg grated, one teaspoonful of ginger, one pound of flour, and one pint of milk; to the eggs, previously well beaten, add one-half the milk, and mix well together; stir in the flour, the spice, fruit and suet, and as much milk as is requisite to reduce the mixture to a plastic consistency, but quite thick. Boil from four to five hours.

